# Membrane potential depolarization causes alterations in neuron arrangement and connectivity in cocultures

**DOI:** 10.1002/brb3.295

**Published:** 2014-11-05

**Authors:** Nurdan Özkucur, Kyle P Quinn, Jin C Pang, Chuang Du, Irene Georgakoudi, Eric Miller, Michael Levin, David L Kaplan

**Affiliations:** 1 Department of Biomedical Engineering, Tufts University4 Colby St., Medford, Massachusetts, 02155; 2 Biology Department, Tufts University200 Boston Avenue, Suite 4600, Medford, Massachusetts, 02155; 3 Department of Electrical and Computer Engineering, Tufts University161 College Avenue, Medford, Massachusetts, 02155; 4 Department of Neuroscience, Tufts University136 Harrison Ave, Boston, Massachusetts

**Keywords:** Automated cluster analysis, cell resting membrane potential, connectivity, cortical neurons, neuron assemblies

## Abstract

**Background:**

The disruption of neuron arrangement is associated with several pathologies. In contrast to action potentials, the role of resting potential (V_mem_) in regulating connectivity remains unknown.

**Methods:**

Neuron assemblies were quantified when their V_mem_ was depolarized using ivermectin (Ivm), a drug that opens chloride channels, for 24 h in cocultures with astrocytes. Cell aggregation was analyzed using automated cluster analysis methods. Neural connectivity was quantified based on the identification of isolated somas in phase-contrast images using image processing. V_mem_ was measured using voltage-sensitive dyes and whole-cell patch clamping. Immunocytochemistry and Western blotting were used to detect changes in the distribution and production of the proteins.

**Results:**

Data show that V_mem_ regulates cortical tissue shape and connectivity. Automated cluster analysis methods revealed that the degree of neural aggregation was significantly increased (0.26 clustering factor vs. 0.21 in controls, *P* ≤ 0.01). The number of beta-tubulin III positive neural projections was also significantly increased in the neural aggregates in cocultures with Ivm. Hyperpolarized neuron cells formed fewer connections (33% at 24 h, *P* ≤ 0.05) compared to control cells in 1-day cultures. Glia cell densities increased (33.3%, *P* ≤ 0.05) under depolarizing conditions.

**Conclusion:**

V_mem_ can be a useful tool to probe neuronal cells, disease tissues models, and cortical tissue arrangements.

## Introduction

Cortical arrangement, including shape and size at the cellular and tissue levels, relates to neural connectivity associated with normal brain development and function (Anstotz et al. 2013; Comin and da Fontoura Costa 2013; Foxworthy et al. 2013). We have used the term ‘neuron arrangement’ as a general term referring to the positional reorganization of cells in respect to each other. Neural assemblies, as first defined by Donald Hebb, are diffuse structures comprising cells in the cortex and diencephalon. Those structures are also capable of acting briefly as a closed system, delivering facilitation to other such systems. Neural assemblies are implicated in complex brain functions such as learning or planning, and accepted as the smallest representatives of a cognitive concept in the brain, as explained in Hebbian theory (Hebb 1949). Neural assemblies are suggested to constitute neural syntaxes that integrate and parse the fundamental assemblies of neural activities (Buzsaki 2010).

Deficits in neuronal organization and connectivity can result in a range of pathologies. Brains of corticobasal syndrome patients have a common degenerating neural network comprised of the motor circuit and primary motor-cortex (Sudmeyer et al. 2012). Corticostriatal connectivity with a precise arrangement of large scale loops has been suggested to play a critical role in neurodevelopmental, neuropsychiatric, and movement disorders (Shepherd 2013). In autism, altered connectivity due to changes in the ratio of distinct types of inhibitory neurons has been reported (Zikopoulos and Barbas 2013). Similarly, profound alterations have been recorded from cortical and subcortical networks in epileptic seizures (Spencer 2002). Changes in brain functional connectivity are detected in patients with Obsessive-Compulsive Disorder and in social anxiety disorder (Fontenelle et al. 2012; Gimenez et al. 2012).

The deformation or volume change in cortical tissue occurs in several neurological diseases. For example, hippocampal shape and volume can predict the onset of dementia in the elderly (Csernansky et al. 2005). In Amyotropic Lateral Sclerosis, a significant thinning due to reduced gray matter has been detected in multiple motor and extramotor cortical areas (d'Ambrosio et al. 2014). Brain morphometric studies have revealed focal abnormalities in several areas of brain, including the cortex, in schizophrenia patients (Davatzikos et al. 2005; Zierhut et al. 2013). Volume loss and cortical thinning was found in the medial temporal lobe of individuals with schizophrenia when compared with control groups. Also correlations between structural measures and memory performance in schizophrenia subjects were demonstrated (Karnik-Henry et al. 2012). Significant variations in shape have been reported in the frontobasal brain region in Obsessive-Compulsive Disorder (Pujol et al. 2011). Reductions in cortical thickness in the left hemisphere and a lack of integrity of white matter is a main contributor to long-term impairment in declarative memory among patients suffering from severe and diffuse traumatic brain injury (Palacios et al. 2013). A recent study verified that cortical thinning is observable/quantifiable and is functionally relevant in blast-related traumatic brain injury patients (Tate et al. 2014). Interestingly, regardless of the disease-induced cause, a decline in cortex volume has also been identified in elderly (Thomann et al. 2013). However, it is still not known which cell types in number, density, or size in the brain are disturbed related to neurological disorders.

Magnetic resonance imaging of clinical subjects is the most common approach used to assess functional brain anatomy. This analytical approaches provides rapid and general diagnosis for clinical purposes (Bernhardt et al. 2013), but lacks spatial resolution at the cellular level, which is important to elucidate the mechanisms of disease and development. In vitro model systems and automated quantitative analysis tools are needed to achieve insight into disease mechanisms and to implement approaches for disease control and therapy.

V_mem_ can regulate cell differentiation as undifferentiated cells exhibit relatively depolarized V_mem_ compared to terminally differentiated cells (Levin 2007). The underlying mechanism for that is the regulation of cell proliferation and differentiation (Sundelacruz et al. 2009). It has long been known that activity of the nervous system during embryogenesis helps regulate differentiation, proliferation, and topology of the nascent CNS (Katz and Shatz 1996; Penn and Shatz 1999; Deisseroth et al. 2004; Young et al. 2012). However, despite the exciting recent data indicating roles for resting potential gradients in embryonic patterning of many organs (Levin et al. 2002; Lange et al. 2011; Levin 2012, 2013; Levin and Stevenson 2012; Beane et al. 2013; Tseng and Levin 2013), the roles of V_mem_ in neural connectivity remain unknown. The objective of this study was to develop a novel approach to mimic and detect abnormalities in cortical tissue arrangement based on changes in the neuron arrangement, as well as shape and size of neural cell assemblies. Ivm is known to change the cellular chloride content. Ivm can change the V_mem_ specifically by binding to Glycine Receptor Chloride Channel alpha-1 subunit. That makes Ivm a specifically targeted and a convenient tool to change the V_mem_ in cells expressing the channel (Sharmeen et al. 2010; Blackiston et al. 2011; Lynagh et al. 2011; Dutertre et al. 2012). We found that Ivm can affect both astrocytes and neurons, as western blot studies revealed that GlyR was produced also in astrocytes. However, the ability of Ivm to affect V_mem_ was in different time windows for neurons and astrocytes. Ivm caused a relatively hyperpolarized V_mem_ in astrocytes alone at 2w and 4w where it reversed neuron V_mem_ (depolarized) at 3 weeks in presence of astrocytes versus the controls (Ozkucur et al., in review). Using intrinsic chloride-dependent V_mem_ shift from depolarizing to hyperpolarizing in developing neurons (Ehrlich et al. 1999), cortical shape and connectivity were controlled with a V_mem_-specific manner using Ivm. The data reveal that changes in gross cortical anatomy can be recapitulated at the cellular level, and suggest V_mem_ as a useful tool to establish in vitro models for both normal and diseased cortex.

## Materials and Methods

E18 rat cerebral cortical tissue was kindly provided every week from Gregory Frost from Dr. Steven Moss's laboratory at Tufts Neuroscience Center. The brain tissue isolation protocol was approved by Tufts University Institutional Animal Care and Use Committee and complies with the NIH Guide for the Care and Use of Laboratory Animals (IACUC # B2011-45).

### Dissociation and plating of rat cortical neurons

Cortices were removed from Hanks Balanced Salt Solution (HBSS) using a 10 mL pipette, and were placed in a pre-warmed 0.25%/0.3% Trypsin/DNase solution in a new 35-mm dish. Cortices were incubated at 37°C for 20 min for enzymatic dissociation of the tissues. The tissue suspension was mixed with pre-warmed trypsin inhibitor solution (1 mg/mL) at 1:1 ratio in a 15 mL tube. The mixture was pipetted up and down <20 times until the tissues were broken up into a homogeneous cell suspension. Dissociated neurons were centrifuged at 1000 rpm for 5 min. The cell pellet was resuspended in 10 mL dissociation buffer (complete NB media + 1:50 glutamate). Cells were counted using a hemocytometer. Cells were seeded on poly-d-lysine (100 *μ*g/mL) coated plates in Neurobasal Media, supplemented with B27, 1% penicillin/streptomycin and 1% glutamax, all purchased from Invitrogen (Carlsbad, CA). Ivm (1 *μ*mol/L, Tocris Bioscience-R&D Systems, Inc., MN) was added to the culture medium to change V_mem_ of the cells. Cell culture media were changed once a week. Each series of experiments constituted the same pool of cortical cells isolated from one rat cerebral cortex. Ninety-six-well plates (Nunc, Rochester, NY), 8-well chambers, and 3-cm petri dishes (Ibidi, LLC, Verona, WI) were used for high-throughput measurements, immunocytochemistry, and connectivity assays, respectively.

### Isolation and culture of rat astroglial cells

Each cortex was taken up with a 10 mL pipette and placed into a 15 mL conical tube containing 5 mL warmed 10% FBS DMEM + 1% penicillin/streptomycin media. Cortices were pipetted up and down <20 times. The homogenized cell suspension was passed through a 40 *μ*m cell strainer into a 50 mL tube. The cell strainer was moved to a new tube and 5 mL of cells were pipetted from a 50 mL conical tube into T-25 flasks. The next day after seeding, the media were replaced with prewarmed fresh 10% FBS DMEM + 1% penicillin/streptomycin. This process was repeated 2–5 days after seeding. After 1 week, the media were removed, cells were rinsed with PBS, and new prewarmed 2% FBS DMEM + 1% penicillin/streptomycin media were added to the cells. Neurons and astrocytes were seeded in 3:1 (3 × 10^5^:1 × 10^5^ cells/mL) ratio for cocultures. Each series of experiments constituted the same pool of astrocytes isolated from cerebral cortices of the offspring of one pregnant rat. Cocultures were maintained in astrocyte media combined with complete neurobasal media at 37°C in a humidified incubator with 5% CO_2_. Medium was changed once in a week. Astrocyte cultures were split 1:5 once a week.

### Cell toxicity assay WST-1

Cell toxicity data were collected from WST-1 assay that measures mitochondrial activity. This assay is based on a tetrazolium salt is cleaved to formazan by the ‘succinate-tetrazolium reductase’ system which belongs to the respiratory chain of the mitochondria and is active only in viable cells (Hoper 1997; Ngamwongsatit et al. 2008). Cultures at 0–4 weeks after plating were incubated for 2 h, at 37°C with WST-1 reagent (1:10 in cell culture medium, Roche). Mitochondrial dehydrogenase activity in vital cells was measured as optical density at 440 nm using multimode multiplate reader (SpectraMax M2, Molecular devices, Sunnyvale, CA) with SoftMax Pro software. After measurement, cells were trypsinized and cell numbers were counted using hemocytometer. Cell viability was represented as optical density (OD) values divided by corresponding cell numbers (N_cell_).

### Immunocytochemistry

Cells were washed once with Ca^2+^- and Mg^2+^-containing PBS (pH 7.4), fixed in 4% formaldehyde/PBS for 30 min at room temperature (RT), permeabilized with 0.03% Triton X-100/PBS (Sigma Aldrich) for 5 min. Primary antibodies were added in 10% FBS/PBS and incubated overnight at 4°C. After washing with PBS, cells were further treated with fluorescence-coupled secondary antibodies in 10% FBS/PBS at 37°C for 30 min. Fluoroshield Mounting Medium With DAPI (Abcam, Cambridge, MA) was used as a mounting solution. The following antibodies were used; rabbit polyclonal Vimentin (Sigma-Aldrich Cat# SAB4503083, RRID:AB_10746985), mouse monoclonal Glial Fibrillary Acidic Protein (GFAP, Sigma-Aldrich Cat# G3893 RRID:AB_477010)), rabbit polyclonal Glycine Receptor Alpha 1 (GLRA1, Abcam Cat# ab475, RRID:AB_304586), mouse monoclonal Synaptophysin (Abcam Cat# ab8049 RRID:AB_2198854), rabbit polyclonal beta III-tubulin (Abcam Cat# ab18207 RRID:AB_444319), anti-mouse Alexa Fluor 568 (Life Technologies Cat# A11004 RRID:AB_10562368), anti-rabbit Alexa Fluor 488 (Molecular Probes Cat# A11008 RRID:AB_143165), and Hoechst 33342 (Invitrogen, Cat# H1399). Images were collected using Leica DM, (Wetzlar, Germany) inverted fluorescence microscope, equipped with a mercury lamp, ebq 100 and a monochrome camera. 20X and 40X objectives were used. DAPI (excitation 360 ± 20 nm and emission 470 ± 20 nm), GFP (excitation 470 ± 20 nm and emission 525 ± 25 nm), and TexasRed (excitation 560 ± 20 nm and emission 645 ± 40 nm) filter sets were used.

### Western blot

The samples were reduced and denatured by boiling each cell lysate in sample buffer at 65°C for 5 min. Samples were loaded into the wells of the SDS-PAGE gel, along with molecular weight markers. Ten *μ*g of total protein was loaded from cell lysate. The gel was run at 80V for 30 min, and then switched to 150V and run 1–2 h. The protein was then transferred from the gel to PVDF membrane using 200 mA current for 2 h.

The membrane was blocked in room temperature for 1 h or overnight at 4°C using 5% blocking solution. The membrane was then incubated with the appropriate dilutions of primary antibody in 5% BSA overnight at 4°C or for 2 h at room temperature. The membrane was washed in PBST three times, 5 min each. Membrane was then incubated with the recommended dilution of labeled secondary antibody in 5% blocking buffer in PBST at room temperature for 1 h. After washing the membrane three times in PBST, the membrane was soaked in Pierce ECL western blotting substrate for 1 min for signal development. The membrane was covered in transparent plastic wrap before for imaging.

### Membrane potential measurement using Di-8-ANEPPS dye

Ratiometric di-8-ANEPPS dye (Invitrogen, Carlsbad, CA) was used to measure the V_mem_ of neurons (N), astrocytes (A) and cocultures (NA) from 0–4 weeks (w) after plating. Cells were washed once with HBSS prior to staining. Di-8-ANEPPS dye was diluted in HBSS to a final concentration of 2 *μ*mol/L and added to the cells (100 *μ*L/well). Cells were incubated at 37°C for 30 min in the dark. The uptake of dye was confirmed under a microscope using a red filter. Cells were washed once with HBSS to remove the excessive dye prior to measurement. Fluorescent intensities were measured using a top read fluorescence multimode multiplate reader (SpectraMax M5, Molecular Devices, Sunnyvale, CA). The relative V_mem_ of the cells was measured as the ratio of readings at two excitation states of the dye (450 nm/510 nm, emission: 640 nm). Plots were generated in excel. *P* values were calculated using Student's t-test.

### Membrane Potential Measurement using whole-cell patch clamping

**Figure 1:**
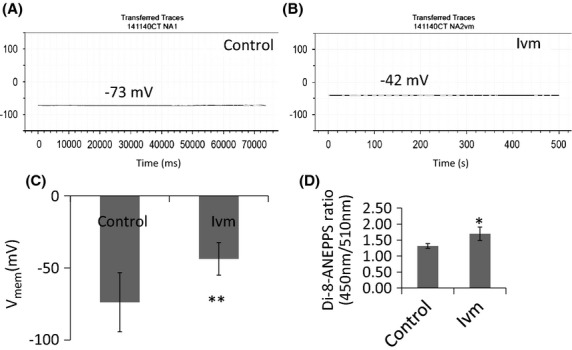
Whole-cell patch clamp recordings and Di-8-ANEPPS measurements from neurons in 4-day cocultures. (A) Single recording from a control (no drug) neuron. (B) Single recording from an ivm-treated neuron. (C) Plot showing the average V_mem_ values recorded from control and ivm-treated cells. **P ≤ 0.01. *N* = 12 (D) Plot showing the average V_mem_ values measured from control (no drug) and ivm-treated neuron cultures. *P ≤ 0.05, *N* = 14.

Cultured rat embryonic day 18 cortical neurons plated on poly-D-lysine-coated 10-mm glass cover slips with or without astrocytes 4–6 days ago were placed inside a recording chamber (P1, Warner Instruments) that contained extracellular solution (140 mmol/L NaCl, 2.8 mmol/L KCl, 2 mmol/L CaCl_2_, 2 mmol/L MgCl_2_, 10 mmol/L HEPES, and 10 mmol/L D-glucose, with pH adjusted to 7.4 with NaOH). Cells were visualized with a Zeiss Axiovert 100 inverted microscope. Membrane potentials were measured at room temperature by whole-cell patch clamp using an Axopatch 1D amplifier (Axon Instruments) in current-clamp mode. Patch electrodes were pulled from 1.5-mm-diameter borosilicate glass capillaries (Sutter) with a Sutter P-87 microelectrode puller, and had 4–6 MΩ resistances when filled with an intracellular solution that contained 140 mmol/L potassium gluconate, 10 mmol/L NaCl, 2 mmol/L MgCl_2_, 10 mmol/L HEPES, 1 mmol/L EGTA, 4 mmol/L MgATP, and 0.3 mmol/L NaGTP, with pH adjusted to 7.3 with KOH. Membrane potential data were filtered by the amplifier-incorporated 4-pole Bessel filter at 2 KHz, and digitized at 5 KHz by a Molecular Devices digitizer (Digidata 1440A) using a Dell Precision 340 computer, with pClamp 10 software (Molecular Devices). V_mem_ quantification based on two methods, di-8-ANEPPS and whole-cell patch clamping, revealed that 1% change in ANEPPS ratio corresponds to 1 mV change in V_mem_ in E18 cortical neurons (Fig.1).

### Clustering, cell area, and density analysis

To quantify the extent to which cells clustered, a Fourier-based analysis of image intensities was utilized through the development of a custom-written Matlab code. This method enabled us to analyze large number of cells immune-labeled for three different cellular components in an automated manner. Images that were collected with different objectives and at different digital resolutions were first resampled to a consistent digital resolution (800 × 600 pixels; 1.6 pixels/*μ*m) that exceeded the diffraction-limit optical resolution of all objectives in an effort to standardize the image data prior to cluster analysis. To facilitate Fourier-based cluster analysis, images were further cropped to a 600 × 600 pixel image by taking the center portion of the original image. A two-dimensional (2D) power spectral density (PSD) map was obtained through a discrete Fourier transform, and the 2D PSD was then radially sampled to produce a single PSD-frequency curve for each image as previously described (Xylas et al. 2012). The PSD-frequency data were log transformed, and a PSD-weighted average spatial frequency (*f*) was then computed using every data point *i*:

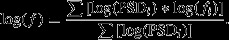


A normalized clustering factor (NCF) was defined from the PSD-weighted average frequency as follows:

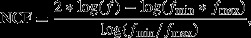


where *f*_min_ and *f*_max_ represent the minimum and maximum spatial frequencies in the PSD curve. In this normalized scale, random (white) noise would produce a NCF = 0, whereas a single large Gaussian distributed clump would correspond to the maximum value of NCF = 1. Of note, PSD-frequency curves demonstrating a power law decay (e.g., a self-similar fractal) would produce an NCF = 0.5. NCF values were obtained from each fluorescence channel in the acquired images.

### Cell counting and area estimation

The number of cells in each image and the average cell area were also approximated through automated image analysis algorithms written in Matlab. Cells were counted by identifying the locations of individual DAPI-stained nuclei. First, pixel intensity values were normalized in the DAPI images to span between 0 and 1. To highlight the location of nuclei, which spanned 12–18 pixels in diameter, two filtered DAPI images were produced through convolutions with 12 and 18 pixel diameter disks. An image of the difference between the two filtered images was produced to highlight nuclei between this 12–18 pixel diameter range. Pixel locations from this filtered image exceeding a value of 0.005 were noted. Next, a Prewitt edge finding filter was applied to the filtered image to identify pixel locations with a high intensity gradient. Objects made of contiguous pixels with intensities exceeding 0.005 and a gradient value exceeding 0.02 were identified, and the locations of individual nuclei were defined by the centroids of each of these objects. These two intensity threshold values for the processed DAPI images were chosen based on their collective ability to provide an accurate identification of nuclei across all image sets. Nuclei were labeled as neurons if the intensity of the green channel (beta III tubulin/Alexa Fluor 488) exceeded the intensity of the red channel (Glial Fibrillary Acidic Protein/Alexa Fluor 568) at that pixel location, whereas astrocytes were defined when the red intensity exceeded the green intensity.

The relative areas covered by astrocytes and neurons were also calculated from the image intensities in each channel. Regions covered by astrocytes were defined by pixel locations that (a) had a red channel intensity that exceeded 10% of the image's maximum intensity and (b) also had a higher red channel intensity than green channel intensity. The neuronal regions were defined by pixel locations at which the green intensity exceeded both 10% of the maximum image intensity and also the red channel intensity at that location. The average area of an individual cell within each image was computed from the total area of a given cell type and the number of nuclei identified and classified as that cell type.

### Connectivity (neural network) analysis

Phase-contrast images collected from control and Ivm-treated cortical neuron cultures were used to analyze V_mem_-induced changes in the neural connectivity. 35-mm-petri dishes with low walls and an imprinted 500 *μ*m relocation grid were used for both culturing and imaging (Ibidi, LLC, Verona, WI). Imprinted grids enabled the relocation of the same cells at different time points during imaging. We focused on analyzing phase-contrast images rather than fluorescence images of the labeled cells. The reason for that was a) to avoid the morphological changes due to the staining process, and b) to be able to work on the most natural state of the neurons as possible in vitro. The somas were manually annotated, and then the isolated somas were identified. We categorized somas as ‘connected’ if they were connected to others by a dendrite. In our case, we used 10 pixels as the distance threshold; usually somas closer than 10 pixels shared a common “halo”, which is an artifact of phase-contrast microscopy. Isolated somas were identified as somas that were neither connected with others by a dendrite nor close in proximity to another soma. As an example, there were 43 somas identified in Fig.4C, which shows one 0.5 × 0.5 mm area (a grid) of the entire dish; the somas are numbered as well as the boundaries of the somas. The very bright areas, which were assumed to be dead cells, were omitted. We identified somas that were connected through dendrites as the first condition of connectivity. Although there were no dendrites among some somas, they were very close to each other and thus we also treated these somas as connected.

## Results

### Mature neurons had increased numbers of projections when their V_mem_ was depolarized

**Figure 2:**
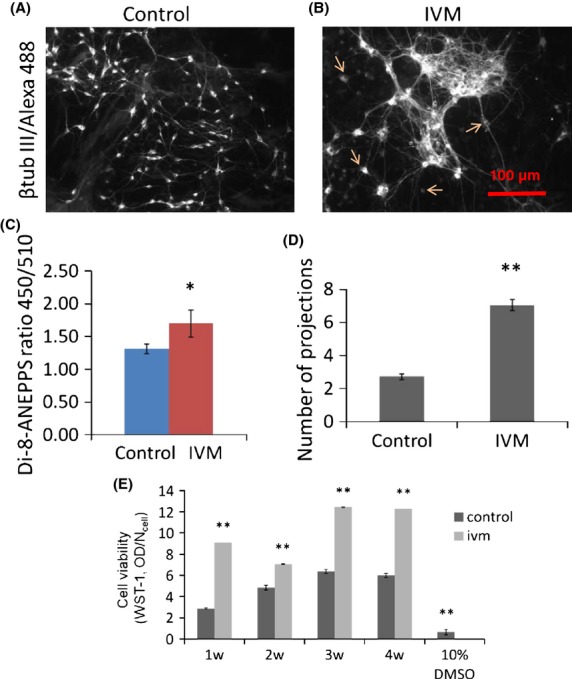
Depolarized cell membrane causes more projections to be formed in mature neurons; 3 week cocultures of rat primary cortical neurons and astrocytes were stained with beta tubulin III and Alexa Fluor 488 to visualize the neurons, (A) under control and (B) Ivm-treated (1 *μ*mol/L, 24 h) conditions. (C) Ivm-caused depolarization of the cell membrane detected using ratiometric dye Di-8-ANEPPS. *N* = 12. (D) The numbers of neural projections of single cells (arrows) were counted. *N* = 18. (E) Cell viability at 1, 2, 3, and 4 weeks in control (no drug) and Ivm-treated cultures. 10% DMSO was used as negative control. *P ≤ 0.05, **P ≤ 0.01. *N* = 18.

The total number of projections emerging from single neurons (arrows) was counted manually in the fluorescent (beta III tubulin/AlexaFluor488) images taken from control (Fig.2A) or Ivm-treated 3w cocultures (Fig.2B). Depolarization of neural V_mem_ was verified by ratiometric measurement of V_mem_-specific dye di-8-ANEPPS in control (no Ivm) and in Ivm-treated 3w cocultures. Accuracy of di-8-ANEPPS dye was verified by whole-cell patch clamp measurements as shown in the methods section. When incubated with 1 *μ*mol/L Ivm for 24 h, the di-8-ANEPPS ratio (450 nm/510 nm) signal measured from cocultures was significantly increased (by 29%), indicating a depolarized V_mem_ (Fig.2C). The V_mem_ of glial cells in homotypic cultures did not change upon Ivm exposure. Differentiated neurons had a significantly increased (133%, *P* ≤ 0.01) number of projections when their V_mem_ was depolarized using 1 *μ*mol/L Ivm for 24 h. Control cells had fewer projections (Fig.2D). Cell toxicity was examined using the WST-1 assay, which measures cell health via readout of mitochondrial activity (Hoper 1997; Ngamwongsatit et al. 2008). We found that 1 *μ*mol/L Ivm was not toxic to E18 cortical cells during 4 weeks in vitro. In contrast, cells treated with the drug exhibited significantly higher mitochondrial activity than cells without the drug (Fig.2E). We conclude that depolarized V_mem_ causes mature neurons to form more membrane projections.

### Change in organization of mature neural assemblies via V_mem_

**Figure 3:**
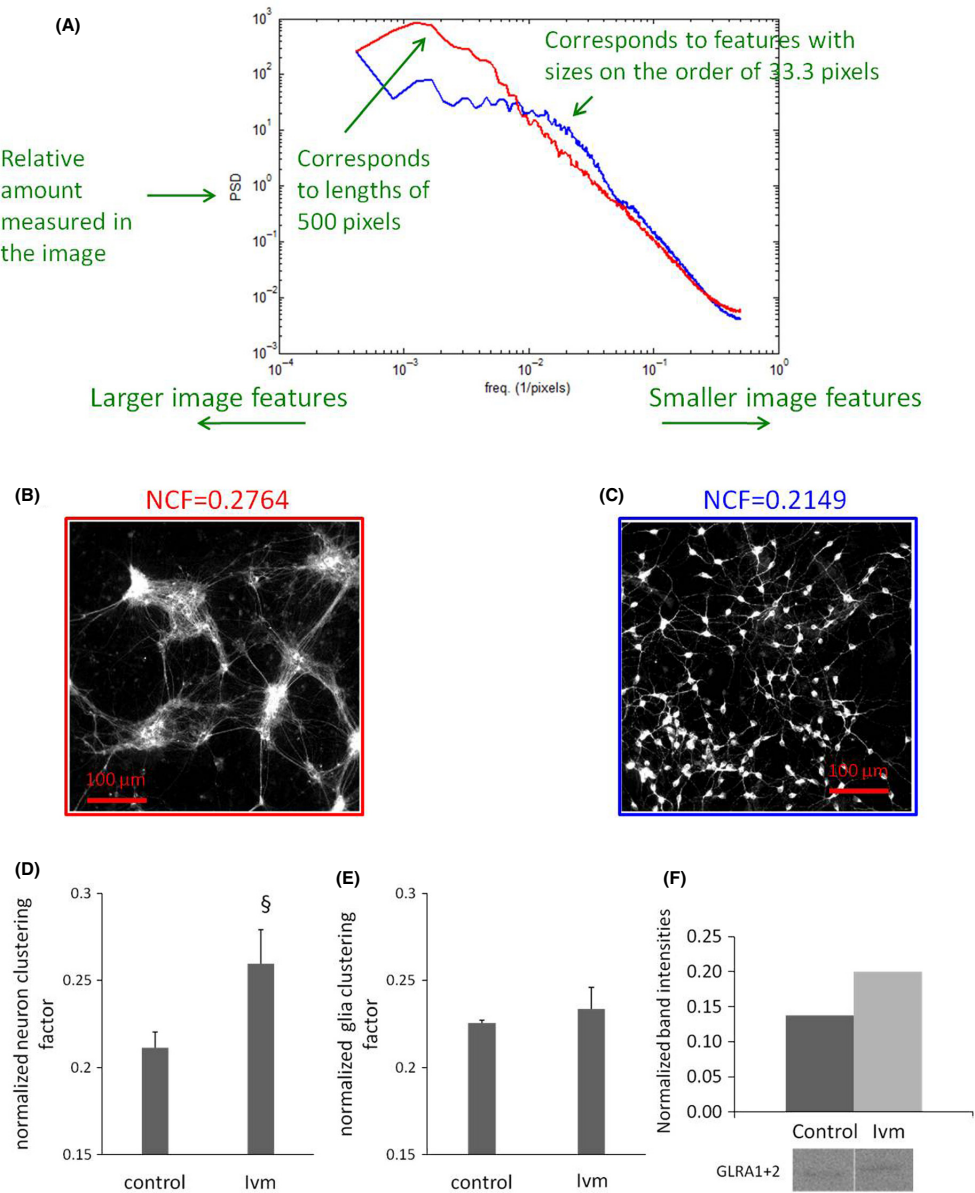
Depolarized cell membrane causes mature neurons assemble into aggregates. (A) Cluster analysis; *y* axis of the plot is power spectral density and, *x* axis is spatial frequency, which has units of pixels^−1^. Red line corresponds to Panel B image and blue to Panel C image. Single-channel images taken from (B) Ivm-treated cells (3 weeks cocultures, 1 *μ*mol/L, 24 h) and (C) from Control (not treated with ivm) are shown here as representative. A large aggregate is equal to one and random organization (no aggregation) is 0. (D) Ivm caused significant neuronal aggregation. (E) A very little glial aggregation was observed (E). §*P ≤ 0.01*. *N* = 18 images, 54,000 cells in total. (F) Western blot data showing increased GLR production in cells with depolarized V_mem_ (Ivm). Band intensities were normalized to one taken as the highest intensity band.

We used a pharmacological strategy (Blackiston et al. 2011; Adams and Levin 2013) targeting native chloride channels to stably depolarize the V_mem_ of cells in culture and to ascertain the effects on cell distribution. The distribution of neural cells was quantified by performing automated cluster analysis on the fluorescent images. Images were taken from 3w cocultures labeled with beta III-tubulin and GFAP for visualizing neurons and astrocytes, respectively. The *y* axis of the plot is power spectral density (PSD), analogous to a histogram frequency count. The PSD was generated by transforming the images to Fourier space as previously described (Xylas et al. 2012). The *x* axis is spatial frequency, which has units of pixels^−1^. An image feature, like a clump of cells that is 100 pixels in diameter would correspond to a frequency of 0.01 pixels^−1^ (Fig.3A). Control cells were distributed mostly sparsely with single cells or very small aggregates of a few cells (Fig.3C). In contrast, mature neurons assembled into aggregates when 3w cocultures were exposed to 1 *μ*mol/L Ivm for 24 h (Fig.3B). A normalized clustering factor was defined based on the PSD-weighted average frequency, in which the largest possible aggregate within the images is equal to 1 and random organization (no aggregation) is 0. Under depolarizing conditions (1 *μ*mol/L Ivm, 24 h), neurons showed significantly greater aggregation by a degree of 0.26 compared to controls (0.21) with no Ivm exposure (Fig.3D). In contrast, glia cell aggregates had no significant difference in their clustering factor between Ivm and control conditions (Fig.3E). We conclude that mature neurons cluster together to form large assemblies when their cell membrane is depolarized. We also examined these assemblies using a calcium pump (SERCA)-opener Thapsigargin (TG) on cell cultures loaded with 2 *μ*mol/L Fluo4-AM and found that the assemblies were functionally connected to each other. Real-time connectivity was recorded as a calcium wave propagating among V_mem_-induced (24 h Ivm) neural assemblies upon TG (20 *μ*mol/L)-induced depolarization. TG was applied on the left side of the dish during recording (Supplementary Movie S1). Glycine receptor (GLR) production increased in cells with depolarized membrane potential, which may be linked to the formation of the assemblies (Fig.3F).

### Depolarized V_mem_ in mature neurons caused an increase in glial cell density

**Figure 4:**
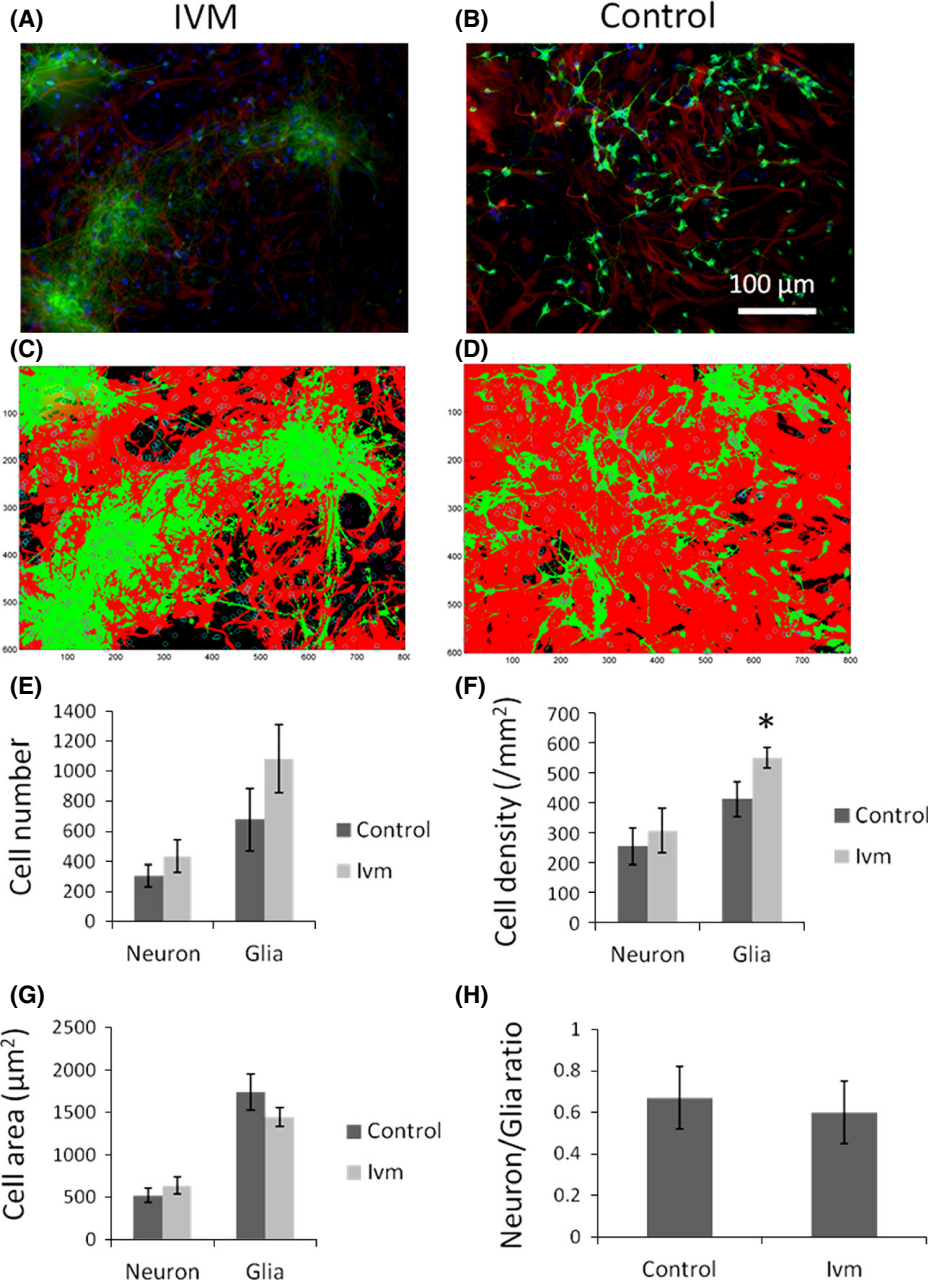
Glial cell density increases when V_mem_ of mature neurons depolarized. 3w cocultures of neurons and astrocytes were visualized using beta tubulin III/AlexaFluor 488 (neurons) and GFAP/AlexaFluor 563 (astrocytes), respectively. DAPI was used as counter stain. (A and B) Original fluorescently labeled images of neuron and glia in cocultures under depolarizing (ivm)- and control conditions. (C and D) Masks of the same images. Cells labeled as neurons based on a greater intensity of green stain relative to red, were circled in magenta. Those cells labeled as glia was circled in cyan. (E) Increased number of neuron and glia cells in ivm-treated samples. (F) Glia cell density increased significantly (*P* ≤ 0.05) due to depolarization. (G) Depolarization increased total area of neural cells while decreasing that of glia cells. (H) A slight reduction is observed in neuron/glia ratio under depolarizing conditions. *N* = 18 images, 54,000 cells in total. *P ≤ 0.05.

Segmentation analysis was performed to locate and quantify the nuclei in fluorescent images from cells labeled with DAPI (small circles). Cells were labeled as neurons based on a greater intensity of green stain relative to red and are circled in magenta. Those cells labeled as glia are circled in cyan. False-colored masks (Fig.4A and B) showing glia and neuron areas were generated from the corresponding original images (Fig.4C and D). Glial and neural cell areas were identified based on red (GFAP/AlexaFluor 563) and green (Beta-III tubulin/AlexaFluor 488) intensity profiles, respectively. In 3w cocultures treated with Ivm (1 *μ*mol/L, 24 h), both neuron (33.6%) and glia (59.7%) cells had greater numbers than controls with no Ivm exposure (Fig.4E). Similarly, both glial (33.3%, *P* ≤ 0.05) and neural (20.4%) cell densities were greater in 3w cocultures under depolarizing conditions (1 *μ*mol/L Ivm, 24 h) compared to their densities in control conditions with no Ivm exposure (Fig.4F). In contrast, divergent changes were detected between neural and glial cell area profiles when neural V_mem_ was depolarized in 3w cocultures. After depolarization, the neurons had an average size of 643 *μ*m^2^, larger than the controls (521 *μ*m^2^), whereas glia had a reduced cell size of 1449 *μ*m^2^ compared to controls (1743 *μ*m^2^). Data revealed that the average size of the neurons increased by 18.9% whereas that of glia declined by 20.3% when the V_mem_ of the neurons was depolarized (Fig.4G). Additionally, the ratio of neuron and glia cell counts decreased slightly (10.4%) in 3w cocultures under depolarizing conditions (Fig.4H). We conclude that a depolarized V_mem_ alters the neuronal microenvironment due to the higher density packing of astrocytes surrounding them.

### Immature neurons establish fewer connections under hyperpolarizing conditions

**Figure 5:**
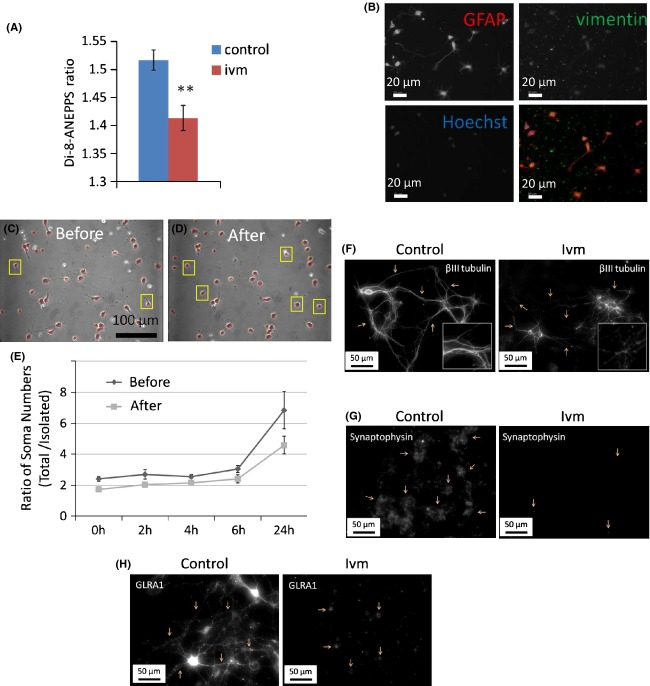
Ivm causes membrane hyperpolarization in 1 day in vitro cultures of E18 rat primary cortical cells. (A) Cell membrane potential was detected colorimetrically using a specific dye Di-8-ANEPPS. **P ≤ 0.01. *N* = 60. (B) 1-day cultures of E18 rat primary cortical cells were stained with GFAP.FITC and vimentin. Alexa 568 was used to characterize the cultures (neural progenitor and stem cells). Cell nuclei were visualized using Hoechst. Connectivity is suppressed in immature neurons under hyperpolarizing conditions in 1 day in vitro cultures of E18 rat cortical cells. Aggregates were excluded. (C and D) The total number of somas versus isolated somas (highlighted in yellow squares) was counted to define connectivity in cells before and after ivm exposure. (E) Ratio of total somas to isolated somas was plotted to represent the changes in neural connectivity under normal (control) and hyperpolarizing conditions (ivm). *N* = 10 microscopic fields from two separate dishes were tracked, and a total of 2500 cells were counted for each control and drug group. (F) Beta-III tubulin labeling showing thin, discontinuous, broken membrane projections (arrows) in ivm-treated cells, whereas control cells have continuous, thick bundles of projections. (G) Synaptophysin labeling showing control cells with large, dense synaptic areas (arrows), whereas lack of those in ivm-treated cells. (H) GLRA labeling showing strong production of the protein along their membrane projections as well as in their somas (arrows), whereas ivm-treated cells have GLRA distribution limited to their somas only. *P* ≤ 0.05 versus controls at each time points.

Neural connectivity was assessed based on the quantification of neurite outgrowth and the establishment of contact between neuron somas. One day cultures of E18 rat primary cortical neurons were assessed under normal physiological (no Ivm)- and depolarizing (after Ivm exposure, 1 *μ*mol/L, 24 h) conditions between 0 and 24 h, with 2 h interval initially. The relative V_mem_ of cells was measured using di-8-ANEPPS dye (Fig.5A). Immature neurons were characterized using specific markers vimentin and GFAP (Fig.5B). Phase-contrast images were collected of the same cells before- and after Ivm exposure at given time points for quantification. Images taken from cells before and after Ivm exposure were shown here (Fig.5C,D). The ratio of the total number of soma to the number of isolated soma was manually counted by two researchers independently.

For imaging, four time points were chosen: 0, 2, 4, 6, and 24 h. The ratio of the total somas to the isolated somas in the images from each time point was obtained. Images were processed, consisting of 10 drug groups and 10 control groups. The ratio of total somas to isolated somas was used as a metric to determine whether Ivm suppressed cell connectivity. Statistical analysis using Student's t-test based on these 100 ratio values (10 × 2 × 5) indicated that there was a significant (*P* ≤ 0.05) difference between the drug and control groups, suggesting the drug dose suppressed cell connectivity.

Data revealed that neural connectivity was significantly suppressed (*P* ≤ 0.05) at each time point used in the study. In Ivm-exposed samples, the ratio of total soma number to isolated soma decreased at 0, 2, 4, 6, and 24 h significantly by 28%, 25%, 16%, 21%, and by 33%, respectively (Fig.5E).

To define synaptic connections, we labeled cells with beta-III tubulin, Synaptophysin and Glycine receptor alpha (GLRA) for axon outgrowth, pre-, and postsynaptic proteins, respectively. Ivm-treated cells had very thin, discontinuous, broken membrane projections (arrows) as labeled with beta-III tubulin,whereas control cells had continuous, thick bundles of projections indicating a well-integrated network of neurons (Fig.5F). Similarly Ivm-treated neurons did not show the presynaptic protein Synaptophysin, whereas control cells had large, dense synaptic areas that were positive for the protein (arrows, Fig.5G). Additionally, postsynaptic protein GLRA was not detected along the projections of Ivm-treated neurons but in somas only, whereas control cells had strong production of GLRA along their membrane projections as well as in their somas (arrows, Fig.5H), revealing that inhibitory synapses were eliminated in Ivm-treated cells. We conclude that membrane potential hyperpolarization caused a decrease in neuron connectivity.

## Discussion

Cortical size and shape have been accepted as an accurate clinical measure for functional brain disorders (Hutton et al. 2008; Abdel Razek et al. 2009; Takao et al. 2010; Prabhakaran et al. 2012; Kuhnt et al. 2013). Many of these disorders can be diagnosed using volumetric measurements, including by functional magnetic resonance imaging or positron emission tomography scanning. However, the underlying mechanisms of these anatomical abnormalities in diseased cortices cannot be gauged at the cellular level via these currently available diagnostic tools. Understanding the cell physiological basis of brain diseases is crucial to specifically characterize the nature of a given disorder. This is needed in order to establish realistic models of neurological diseases in the laboratory, which can then be employed for diagnostic and therapeutic purposes. In this study, we show that V_mem_ can change cortical tissue arrangement, shape, size, and neural connectivity in vitro. We observed that mature cortical neurons aggregated together and formed assemblies when their V_mem_ was depolarized. These assemblies varied in size ranging from 5–10 cells to 40–50 cells in vitro. Hypotheses for the molecular signatures of neuron assemblies (Yagi 2012) are mostly characterized based on firing patterns (Harris 2005). Membrane potential regulation of cell connectivity is a new mechanism in the field. A few pathways have been reported to imply the formation of neural circuit assemblies in vertebrates; Wnt and Semaphorin signaling (Salinas and Zou 2008; Yoshida 2012), gap junctional proteins (Baker and Macagno 2014), and Glycine receptor signaling (Xu and Tian 2008). We found that GLR production increased when neural V_mem_ was depolarized (Fig.2F) indicating that there was an enhancement in neural connectivity due to V_mem_. Several diseases have already been linked to the disturbance in neural assemblies such as Alzheimer's and Epilepsy. Muldoon et al. showed that multiple functional clusters of spatially localized neurons comprise epileptic networks, and that network events are composed of the coactivation of variable subsets of these clusters (Feldt Muldoon et al. 2013). It has been also shown that Alzheimer's disease affects specific and connected neural networks via synaptic terminals affecting brain connectivity well before neuronal loss and compartmental atrophy (D'Amelio and Rossini 2012). Moreover, an abnormal connectivity and impaired cognition was found in Hyperekplexia and in Stiff Person Syndrome. These diseases are caused by hereditary mutations resulting in dysfunction of the Glycine receptor channel (alpha1 and beta subunits) that form inhibitory synapses in the nervous system. Therefore, impairment in glycinergic synapse function causes an imbalance between excitatory and inhibitory function of a neural network (Brune et al. 1996; McKeon et al. 2013; Bode and Lynch 2014). Neuron assemblies were formed as a result of V_mem_ depolarization in this study. This conclusion is also consistent with in vivo data on the role of V_mem_ in developmental pattern formation (Levin et al. 2002; Lobikin et al. 2012; Chernet and Levin 2013; Tseng and Levin 2013). Additionally, it is known that neural assemblies in large circuits are regulated by neural firing in vivo (Buzsaki and Watson 2012; Huyck and Passmore 2013). Recently, a new study suggested that circuit assemblies can be guided by neurogenesis and neural migration during development (Gao et al. 2013). In this study, we quantified single somas in dense cultures of neural networks and found that the average number of projections in mature cortical neurons increased from 3 to 7 under depolarizing conditions. This result indicates that depolarized V_mem_ enhanced the wiring in mature neurons. Membrane depolarization is maintained over 3 weeks of the chronic treatments in our study. Ivm is an agonist of Glycine Receptor Chloride Channel and it leads to membrane depolarization via changing the intracellular chloride concentration. However, the true effector is the changes in resting membrane potential (V_mem_), not the chloride ion flows per se. In another study, we could rescue the downstream effects of Ivm depolarization such as mitochondrial transmembrane potential changes by blocking the gap junctions. This demonstrates that the V_mem_ itself, not specific ion flux, controls the downstream effects (Ozkucur et al., in review).

In immature neurons, however, connectivity was significantly suppressed under hyperpolarizing conditions due to decreased rates of neurite outgrowth resulting in less cell–cell contact, as assessed by calculating the ratio between total and isolated somas. This result showed that V_mem_ regulated the degree of connectivity and also the development of this connectivity during the first weeks of neuron growth in vitro. Whole-brain network organization relates to cell densities in rodents (French and Pavlidis 2011; French et al. 2011). In the monkey brain, cell density in distinct regions can predict the laminar-specificity of connectivity between two regions (Barbas et al. 2005). A recent report also suggested the presence of a combinatorial effect of modular architectures in network organization relates to the nonuniformity of cell densities in the monkey brain (Shimono 2013). Interestingly, depolarization of V_mem_ in mature neurons caused a significant increase in glia cell density along with a decrease in glial cell area in this study, whereas neural cell area increased slightly. It is known that changes in both the cell numbers and size of neuron and glia occur during postnatal brain development (Bandeira et al. 2009) as well as in neurological diseases (Cotter et al. 2001). It has been reported that during the first postnatal week in rats, brain growth relates mainly to increased numbers of neurons of larger average size (Lyck et al. 2007; Bandeira et al. 2009). In the second and third weeks, this process correlates with increased numbers of glial cells and the elimination of 60% of the neurons (Haddara 1956; Brizzee 1964; Bandeira et al. 2009).

The present data originate from 3w cocultures. Therefore, increased glial density under depolarizing conditions (Ivm) detected in vitro might be relevant to gliogenesis that occurs mostly during the second and third weeks of postnatal brain development in rats (Sauvageot and Stiles 2002). In contrast, glia cell size decreased and neuron size increased, whereas the number of both cell types increased under depolarized conditions in 3w cocultures. A parallel increase in neuronal and glial cell density has been shown, for example, in schizophrenia, due to tighter cell packing as a consequence of reduced interneuronal neuropils, rather than as an indication of increased glial cell number (Selemon et al. 1995; Rajkowska et al. 1998). In this study, however, depolarization-induced increased glial cell density was found, and a decreased glial cell area coincided with an increase in glia cell number. Changing V_mem_ has resulted in common phenotypes of cortical volume and neuron networking, as reported in several neurological disorders (Selemon et al. 1995; Cotter et al. 2001; Pujol et al. 2011; d'Ambrosio et al. 2014), in traumatic brain injuries (Selemon et al. 1995; Tate et al. 2014) and in aging (Csernansky et al. 2005). Therefore, we conclude that V_mem_ can be used as a tool to investigate the formation of neural assemblies, establish neurological disease models and to study neural connectivity in vitro. Moreover, our data shed light on a new factor – slow changes in ion channel activity – that may underlie aspects of complex patterning of the embryonic nervous system. These observations can provide a beneficial platform for drug screening studies relevant to a broad spectrum of neurological disorders and may suggest novel strategies for regenerative medicine of the nervous system.

## Supporting Information

**Movie S1.** Real-time connectivity of neural assemblies. Calcium wave is propagating among V_mem_-induced (24 h Ivm) neural assemblies upon Thapsigargin (TG, 20 *μ*mol/L)-induced depolarization. TG, a intracellular calcium pump (SERCA) opener, was applied on the left side of the dish during recording. Cells were loaded with 2 *μ*mol/L of Fluo4-AM in HBSS. *N* = 3.

## References

[b1] Abdel Razek AA, Kandell AY, Elsorogy LG, Elmongy A, Basett AA (2009). Disorders of cortical formation: MR imaging features. AJNR Am. J. Neuroradiol.

[b2] Adams DS, Levin M (2013). Endogenous voltage gradients as mediators of cell-cell communication: strategies for investigating bioelectrical signals during pattern formation. Cell Tissue Res.

[b3] Anstotz M, Cosgrove KE, Hack I, Mugnaini E, Maccaferri G, Lubke JH (2013). Morphology, input-output relations and synaptic connectivity of Cajal-Retzius cells in layer 1 of the developing neocortex of CXCR4-EGFP mice. Brain Struct. Funct.

[b4] Baker MW, Macagno ER (2014). Control of neuronal morphology and connectivity: emerging developmental roles for gap junctional proteins. FEBS Lett.

[b5] Bandeira F, Lent R, Herculano-Houzel S (2009). Changing numbers of neuronal and non-neuronal cells underlie postnatal brain growth in the rat. Proc. Natl Acad. Sci. USA.

[b6] Barbas H, Medalla M, Alade O, Suski J, Zikopoulos B, Lera P (2005). Relationship of prefrontal connections to inhibitory systems in superior temporal areas in the rhesus monkey. Cereb. Cortex.

[b7] Beane WS, Morokuma J, Lemire JM, Levin M (2013). Bioelectric signaling regulates head and organ size during planarian regeneration. Development.

[b8] Bernhardt BC, Hong S, Bernasconi A, Bernasconi N (2013). Imaging structural and functional brain networks in temporal lobe epilepsy. Front. Hum. Neurosci.

[b9] Blackiston D, Adams DS, Lemire JM, Lobikin M, Levin M (2011). Transmembrane potential of GlyCl-expressing instructor cells induces a neoplastic-like conversion of melanocytes via a serotonergic pathway. Dis. Model Mech.

[b10] Bode A, Lynch JW (2014). The impact of human hyperekplexia mutations on glycine receptor structure and function. Mol. Brain.

[b11] Brizzee KR (1964). Effects of single and fractionated doses of total body X-irradiation in utero on growth of the brain and its parts. Nature.

[b12] Brune W, Weber RG, Saul B, von Knebel Doeberitz M, Grond-Ginsbach C, Kellerman K (1996). A GLRA1 null mutation in recessive hyperekplexia challenges the functional role of glycine receptors. Am. J. Hum. Genet.

[b13] Buzsaki G (2010). Neural syntax: cell assemblies, synapsembles, and readers. Neuron.

[b14] Buzsaki G, Watson BO (2012). Brain rhythms and neural syntax: implications for efficient coding of cognitive content and neuropsychiatric disease. Dialogues Clin. Neurosci.

[b15] Chernet BT, Levin M (2013). Transmembrane voltage potential is an essential cellular parameter for the detection and control of tumor development in a Xenopus model. Dis. Model Mech.

[b16] Comin CH, da Fontoura Costa L (2013). Shape, connectedness and dynamics in neuronal networks. J. Neurosci. Methods.

[b17] Cotter D, Mackay D, Landau S, Kerwin R, Everall I (2001). Reduced glial cell density and neuronal size in the anterior cingulate cortex in major depressive disorder. Arch. Gen. Psychiatry.

[b18] Csernansky JG, Wang L, Swank J, Miller JP, Gado M, McKeel D (2005). Preclinical detection of Alzheimer's disease: hippocampal shape and volume predict dementia onset in the elderly. Neuroimage.

[b19] d'Ambrosio A, Gallo A, Trojsi F, Corbo D, Esposito F, Cirillo M (2014). Frontotemporal cortical thinning in amyotrophic lateral sclerosis. AJNR Am. J. Neuroradiol.

[b20] D'Amelio M, Rossini PM (2012). Brain excitability and connectivity of neuronal assemblies in Alzheimer's disease: from animal models to human findings. Prog. Neurobiol.

[b21] Davatzikos C, Shen D, Gur RC, Wu X, Liu D, Fan Y (2005). Whole-brain morphometric study of schizophrenia revealing a spatially complex set of focal abnormalities. Arch. Gen. Psychiatry.

[b22] Deisseroth K, Singla S, Toda H, Monje M, Palmer TD, Malenka RC (2004). Excitation-neurogenesis coupling in adult neural stem/progenitor cells. Neuron.

[b23] Dutertre S, Becker CM, Betz H (2012). Inhibitory glycine receptors: an update. J. Biol. Chem.

[b24] Ehrlich I, Lohrke S, Friauf E (1999). Shift from depolarizing to hyperpolarizing glycine action in rat auditory neurones is due to age-dependent Cl- regulation. J. Physiol.

[b25] Feldt Muldoon S, Soltesz I, Cossart R (2013). Spatially clustered neuronal assemblies comprise the microstructure of synchrony in chronically epileptic networks. Proc. Natl Acad. Sci. USA.

[b26] Fontenelle LF, Harrison BJ, Pujol J, Davey CG, Fornito A, Bora E (2012). Brain functional connectivity during induced sadness in patients with obsessive-compulsive disorder. J. Psychiatry Neurosci.

[b27] Foxworthy WA, Clemo HR, Meredith MA (2013). Laminar and connectional organization of a multisensory cortex. J. Comp. Neurol.

[b28] French L, Pavlidis P (2011). Relationships between gene expression and brain wiring in the adult rodent brain. PLoS Comput. Biol.

[b29] French L, Tan PP, Pavlidis P (2011). Large-scale analysis of gene expression and connectivity in the rodent brain: insights through data integration. Front. Neuroinform.

[b30] Gao P, Sultan KT, Zhang XJ, Shi SH (2013). Lineage-dependent circuit assembly in the neocortex. Development.

[b31] Gimenez M, Pujol J, Ortiz H, Soriano-Mas C, Lopez-Sola M, Farre M (2012). Altered brain functional connectivity in relation to perception of scrutiny in social anxiety disorder. Psychiatry Res.

[b32] Haddara M (1956). A quantitative study of the postnatal changes in the packing density of the neurons in the visual cortex of the mouse. J. Anat.

[b33] Harris KD (2005). Neural signatures of cell assembly organization. Nat. Rev. Neurosci.

[b34] Hebb DO (1949). New York Wiley.

[b35] Hoper J (1997). Spectrophotometric in vivo determination of local mitochondrial metabolism by use of a tetrazolium salt. Physiol. Meas.

[b36] Hutton C, De Vita E, Ashburner J, Deichmann R, Turner R (2008). Voxel-based cortical thickness measurements in MRI. Neuroimage.

[b37] Huyck CR, Passmore PJ (2013). A review of cell assemblies. Biol. Cybern.

[b38] Karnik-Henry MS, Wang L, Barch DM, Harms MP, Campanella C, Csernansky JG (2012). Medial temporal lobe structure and cognition in individuals with schizophrenia and in their non-psychotic siblings. Schizophr. Res.

[b39] Katz LC, Shatz CJ (1996). Synaptic activity and the construction of cortical circuits. Science.

[b40] Kuhnt D, Bauer MH, Ganslandt O, Nimsky C (2013). Functional imaging: where do we go from here?. J. Neurosurg. Sci.

[b41] Lange C, Prenninger S, Knuckles P, Taylor V, Levin M, Calegari F (2011). The H(+) vacuolar ATPase maintains neural stem cells in the developing mouse cortex. Stem Cells Dev.

[b42] Levin M (2007). Large-scale biophysics: ion flows and regeneration. Trends Cell Biol.

[b43] Levin M (2012). Molecular bioelectricity in developmental biology: new tools and recent discoveries: control of cell behavior and pattern formation by transmembrane potential gradients. BioEssays.

[b44] Levin M (2013). Reprogramming cells and tissue patterning via bioelectrical pathways: molecular mechanisms and biomedical opportunities. Wiley Interdiscip. Rev. Syst. Biol. Med.

[b45] Levin M, Stevenson CG (2012). Regulation of cell behavior and tissue patterning by bioelectrical signals: challenges and opportunities for biomedical engineering. Annu. Rev. Biomed. Eng.

[b46] Levin M, Thorlin T, Robinson KR, Nogi T, Mercola M (2002). Asymmetries in H+/K+-ATPase and cell membrane potentials comprise a very early step in left-right patterning. Cell.

[b47] Lobikin M, Chernet B, Lobo D, Levin M (2012). Resting potential, oncogene-induced tumorigenesis, and metastasis: the bioelectric basis of cancer in vivo. Phys. Biol.

[b48] Lyck L, Kroigard T, Finsen B (2007). Unbiased cell quantification reveals a continued increase in the number of neocortical neurones during early post-natal development in mice. Eur. J. Neurosci.

[b49] Lynagh T, Webb TI, Dixon CL, Cromer BA, Lynch JW (2011). Molecular determinants of ivermectin sensitivity at the glycine receptor chloride channel. J. Biol. Chem.

[b50] McKeon A, Martinez-Hernandez E, Lancaster E, Matsumoto JY, Harvey RJ, McEvoy KM (2013). Glycine receptor autoimmune spectrum with stiff-man syndrome phenotype. JAMA Neurol.

[b51] Ngamwongsatit P, Banada PP, Panbangred W, Bhunia AK (2008). WST-1-based cell cytotoxicity assay as a substitute for MTT-based assay for rapid detection of toxigenic Bacillus species using CHO cell line. J. Microbiol. Methods.

[b501] Özkucur N, He J, Levin M, Kaplan DL (2014).

[b52] Palacios EM, Sala-Llonch R, Junque C, Fernandez-Espejo D, Roig T, Tormos JM (2013). Long-term declarative memory deficits in diffuse TBI: correlations with cortical thickness, white matter integrity and hippocampal volume. Cortex.

[b53] Penn AA, Shatz CJ (1999). Brain waves and brain wiring: the role of endogenous and sensory-driven neural activity in development. Pediatr. Res.

[b54] Prabhakaran V, Nair VA, Austin BP, La C, Gallagher TA, Wu Y (2012). Current status and future perspectives of magnetic resonance high-field imaging: a summary. Neuroimaging Clin. N. Am.

[b55] Pujol J, Soriano-Mas C, Gispert JD, Bossa M, Reig S, Ortiz H (2011). Variations in the shape of the frontobasal brain region in obsessive-compulsive disorder. Hum. Brain Mapp.

[b56] Rajkowska G, Selemon LD, Goldman-Rakic PS (1998). Neuronal and glial somal size in the prefrontal cortex: a postmortem morphometric study of schizophrenia and Huntington disease. Arch. Gen. Psychiatry.

[b57] Salinas PC, Zou Y (2008). Wnt signaling in neural circuit assembly. Annu. Rev. Neurosci.

[b58] Sauvageot CM, Stiles CD (2002). Molecular mechanisms controlling cortical gliogenesis. Curr. Opin. Neurobiol.

[b59] Selemon LD, Rajkowska G, Goldman-Rakic PS (1995). Abnormally high neuronal density in the schizophrenic cortex. A morphometric analysis of prefrontal area 9 and occipital area 17. Arch. Gen. Psychiatry.

[b60] Sharmeen S, Skrtic M, Sukhai MA, Hurren R, Gronda M, Wang X (2010). The antiparasitic agent ivermectin induces chloride-dependent membrane hyperpolarization and cell death in leukemia cells. Blood.

[b61] Shepherd GM (2013). Corticostriatal connectivity and its role in disease. Nat. Rev. Neurosci.

[b62] Shimono M (2013). Non-uniformity of cell density and networks in the monkey brain. Sci. Rep.

[b63] Spencer SS (2002). Neural networks in human epilepsy: evidence of and implications for treatment. Epilepsia.

[b64] Sudmeyer M, Pieperhoff P, Ferrea S, Krause H, Groiss SJ, Elben S (2012). Longitudinal deformation-based morphometry reveals spatio-temporal dynamics of brain volume changes in patients with corticobasal syndrome. PLoS ONE.

[b65] Sundelacruz S, Levin M, Kaplan DL (2009). Role of membrane potential in the regulation of cell proliferation and differentiation. Stem Cell Rev.

[b66] Takao H, Abe O, Ohtomo K (2010). Computational analysis of cerebral cortex. Neuroradiology.

[b67] Tate DF, York GE, Reid MW, Cooper DB, Jones L, Robin DA (2014). Preliminary findings of cortical thickness abnormalities in blast injured service members and their relationship to clinical findings. Brain Imaging Behav.

[b68] Thomann PA, Wustenberg T, Nolte HM, Menzel PB, Wolf RC, Essig M (2013). Hippocampal and entorhinal cortex volume decline in cognitively intact elderly. Psychiatry Res.

[b69] Tseng A, Levin M (2013). Cracking the bioelectric code: probing endogenous ionic controls of pattern formation. Commun. Integr. Biol.

[b71] Xu HP, Tian N (2008). Glycine receptor-mediated synaptic transmission regulates the maturation of ganglion cell synaptic connectivity. J. Comp. Neurol.

[b72] Xylas J, Quinn KP, Hunter M, Georgakoudi I (2012). Improved Fourier-based characterization of intracellular fractal features. Opt. Express.

[b73] Yagi T (2012). Molecular codes for neuronal individuality and cell assembly in the brain. Front. Mol. Neurosci.

[b74] Yoshida Y (2012). Semaphorin signaling in vertebrate neural circuit assembly. Front. Mol. Neurosci.

[b75] Young SZ, Taylor MM, Wu S, Ikeda-Matsuo Y, Kubera C, Bordey A (2012). NKCC1 knockdown decreases neuron production through GABA(A)-regulated neural progenitor proliferation and delays dendrite development. J. Neurosci.

[b76] Zierhut KC, Grassmann R, Kaufmann J, Steiner J, Bogerts B, Schiltz K (2013). Hippocampal CA1 deformity is related to symptom severity and antipsychotic dosage in schizophrenia. Brain.

[b77] Zikopoulos B, Barbas H (2013). Altered neural connectivity in excitatory and inhibitory cortical circuits in autism. Front. Hum. Neurosci.

